# Association Between Periodontal Health Knowledge and Oral Hygiene Practices in a National Cohort of Thai Adults

**DOI:** 10.1002/cre2.70297

**Published:** 2026-01-26

**Authors:** Mana Naratippakorn, Paswach Wiriyakijja, Dissara Akkarasrisawad, Nirinya Raoprajong, Pirada Itthipolchai, Vivat Thongchotchat, Pimchanok Sutthiboonyapan

**Affiliations:** ^1^ Private practice Asavanant Dental Clinic Bangkok Thailand; ^2^ Department of Oral Medicine, Faculty of Dentistry Chulalongkorn University Bangkok Thailand; ^3^ Avatar Biotechnologies for Oral Health and Healthy Longevity Research Unit Chulalongkorn University Bangkok Thailand; ^4^ Center of Excellence in Precision Medicine and Digital Health Chulalongkorn University Bangkok Thailand; ^5^ Faculty of Dentistry Chulalongkorn University Bangkok Thailand; ^6^ Department of Periodontology, Faculty of Dentistry Chulalongkorn University Bangkok Thailand; ^7^ Center of Excellence in Periodontal Disease and Dental Implant Chulalongkorn University Bangkok Thailand

**Keywords:** knowledge, oral hygiene, periodontal health, practice

## Abstract

**Objectives:**

Periodontitis is a common oral disease that affects populations globally, however, awareness of periodontal disease prevention remains limited. Since periodontal health knowledge may influence oral hygiene behaviors, which are essential for disease prevention and long‐term maintenance, this study aimed to assess periodontal health‐related knowledge and its association with oral hygiene practices among an adult cohort in Thailand using the validated Adult Periodontal Health Knowledge and Belief Questionnaire.

**Material and Methods:**

A cross‐sectional survey of Thai adults (≥ 18 years) was conducted from December 2023 to January 2024. The questionnaire captured demographics, oral hygiene practices (brushing and interdental cleaning), and periodontal health knowledge (ALPHA‐K). Descriptive statistics, non‐parametric tests, exploratory factor analysis, and multiple linear regression were used.

**Results:**

A total of 1770 participants completed the survey. The mean ALPHA‐K score was 11.83 (2.88), which represents 59.2% of total score. While most recognized that plaque as the cause of gum disease, and smoking and diabetes as risk factors, misconceptions persisted (e.g., floss only removes food; tooth loss is inevitable with ageing; fluoride prevents gum disease). Higher periodontal health knowledge was associated with favorable flossing practices, brushing practices (longer brushing duration, extra‐soft/soft bristles, manual toothbrush use) and higher education, higher income, and urban residence.

**Conclusions:**

A significant knowledge deficit regarding periodontal health exists in this Thai cohort, particularly among rural residents, individuals with lower income, and those reporting inadequate brushing duration or preference for hard bristle brushes or electric toothbrushes in Thailand. Disseminating accurate information on periodontal health and disease is essential to enhance self‐care practices and overall periodontal health outcomes.

**Public Health Relevance:**

Thai adults, particularly those with lower education levels and living in rural areas, demonstrated considerable gaps in periodontal health knowledge. Addressing these gaps through tailored educational interventions is important for improving population‐level periodontal health outcomes.

## 1 Introduction

Periodontitis is a chronic multifactorial inflammatory disease affecting the supporting structures of the teeth, primarily caused by dysbiotic plaque biofilms (Hajishengallis 2014). Its progression is influenced by local, systemic host, and environmental factors (Genco and Borgnakke 2013). If left untreated, periodontitis can lead to tooth loss and compromise oral function, appearance and overall quality of life (Orlandi et al. 2022). Globally, more than 800 million people are affected by severe periodontitis, and approximately one‐fifth of Thai adults are diagnosed with the condition (Bernabe et al. 2020; Niyomsilp et al. 2024). These statistics highlight the importance of effective prevention and early disease detection as public health priorities.

Previous studies in Thailand suggest that awareness of periodontal disease exists but remains uneven, particularly regarding disease etiology, risk factors, and appropriate preventive practices. While basic oral health is relatively common, misconceptions and suboptimal oral hygiene behaviors continue to be reported among Thai adults (Suthasinee Samanchat 2022). Within the knowledge, attitude and practice framework, knowledge is regarded as the pivotal driver of practice. Identifying gaps in knowledge and prevailing practice patterns in a populace provides a sound basis for tailored interventions that foster positive behavioral changes and ultimately improve periodontal health outcomes (Zhao and Wu 2024).

To support such efforts, the Adult Periodontal Health Knowledge and Belief Questionnaire (ALPHABET) was recently developed and validated in Thailand (Bodhidatta et al. 2025). The instrument comprises two domains: periodontal health knowledge (ALPHA‐K) and periodontal health belief (ALPHA‐B). Validation demonstrated robust reliability and construct validity, with patients who had undergone periodontal treatment scoring significantly higher scores than new patients. Despite this, population‐level data on periodontal health knowledge using the validated ALPHA K remain scarce.

The aim of the present study therefore was to employ ALPHA‐K in a large national cohort of Thai adults to quantify periodontal health knowledge, and to examine its association with oral hygiene practices. We hypothesized that a significant positive association exists between periodontal health knowledge (as measured by the ALPHA‐K score) and the adoption of optimal oral hygiene practices in a cohort of Thai adults. Our work, for the first time in a larger Thai cohort, addresses a critical evidence gap and provides insights that inform future periodontal health education and public health interventions in Thailand.

## 2 Methods

### 2.1 Study Design

This was a cross‐sectional, self‐administered online survey of Thai adults (≥ 18 years) able to read Thai and willing to participate. Participants were recruited through an open, nationwide online survey randomly distributed across Thailand via electronic platforms. Data were collected using Google Forms from December 2023 to January 2024.

Eligibility criteria included adults ages ≥ 18 years who could read Thai and self‐reported current residence in Thailand. Individual younger than 18 years and those who did not complete the questionnaire were excluded. The survey was openly disseminated and no individual contact lists or online addresses were collected to ensure anonymity and comply with ethical standards.

Ethical approval was obtained from the Human Research Ethics Committee, Faculty of Dentistry, Chulalongkorn University (HREC‐DCU 2023‐123), in accordance with the Declaration of Helsinki. Electronic informed consent was secured before participation. The questionnaires were structured into three sections:

*Section I:* Demographic data

This section encompassed demographic information such as age, gender, education, employment status, monthly income and sufficiency and place of residence (urban/rural).

*Section II:* Health‐related behavior

This section assessed participants’ smoking status and alcohol use.

*Section III:* Oral hygiene practices

Participants provided details on dental visiting pattern; their frequency and duration of toothbrushing; brushing technique; which was described using simple, non‐technical language rather than by name; toothbrush type (manual/electric) and bristle texture; and interdental cleaning practices (device type, frequency, coverage, and flossing technique).

*Section IV:* Periodontal health Knowledge (ALPHA‐K)

This section comprised 20 true/false items on causes, contributing factors, signs and symptoms, prevention, and treatment of periodontal disease. Correct responses scored one point, yielding a total possible score of 0–20.

### 2.2 Statistical Analyses

Analyses were conducted using SPSS version 26 (SPSS Inc., Chicago, IL, USA). Data distribution was assessed using the Kolmogorov–Smirnov test. Non‐parametric tests (Mann–Whitney *U* for two groups; Kruskal–Wallis with post‐hoc pairwise tests for multiple groups) were used as appropriate, with significance set at *α* = 0.05.

Descriptive statistics, including mean, standard deviation, median, maximum and minimum values, first quartile (Q1), third quartile (Q3), and interquartile range (IQR), were summarized. To reduce dimensionality and identify latent constructs underlying oral hygiene practices, exploratory factor analysis (EFA) with principal component extraction and varimax rotation was performed. Sampling adequacy was confirmed using the Kaiser–Meyer–Olkin (KMO) statistic and Bartlett's test of sphericity.

Factors with eigen values ≥ 1 were retained, and items with loadings ≥ 0.40 were considered meaningful. Participant‐level factor scores were calculated and simultaneously entered as independent variables in multiple linear regression models, with the total ALPHA‐K score as the dependent variable. This approach enabled evaluation of the independent associations of distinct practice domains (e.g., flossing, brushing/economic context, age/education) with knowledge outcomes.

### 2.3 Sample Size Calculation

For multiple linear regression, Green's formula (*N* ≥ 50 + 8 m) was applied, where *m* is the number of predictors (Green 1991). With 21 predictors, the minimum desirable sample size was 218. Our achieved sample of 1770 participants far exceeded this threshold, ensuring robust precision and statistical power.

## 3 Results

### 3.1 Demographic Characteristics of the Cohort

The study cohort comprised 1770 Thai adults with a mean age of 35.1 (11.6) years (range 18–79 years) while 62.7% were females. More than half were unmarried, and held a bachelor's degree, while 54.7% resided in rural areas. The majority were employed full‐time, with monthly income between 10,001 and 30,000 THB (approximately 300–950 USD). Full demographic data are presented in Table 1.

**Table 1:** Demographic data and health related behaviors of the participants (*n* = 1770).

Variables	*n* (%)	ALPHA‐K scores (total = 20)	
		Mean (SD)	*p*‐value^d^
**Demographic data**			
**Age**			
< 40 years	1227 (69.3%)	11.64 (2.82)^a^	< 0.001**
40–59 years	460 (26.0%)	12.35 (2.92)^a^	
≥ 60 years	83 (4.7%)	11.77 (3.29)	
**Gender**			
Men	599 (33.8%)	11.55 (2.75)^a^	< 0.001**
Women	1109 (62.7%)	12.02 (2.94)^a^	
Unspecified	62 (3.5%)	11.24 (2.97)	
**Education**			
Up to high school	201 (11.4%)	11.71 (2.90)^a^	< 0.001**
Bachelor's degree	1134 (75.4%)	11.69 (2.78)^b^	
Postgraduate degree	235 (13.3%)	12.73 (3.27)^a,b^	
**Place of living**			
Urban	802 (45.3%)	12.32 (3.05)	< 0.001**
Rural	968 (54.7%)	11.43 (2.67)	
**Job status**			
Work	1134 (75.4%)	11.91 (2.90)	0.529
Unemployed	81 (4.6%)	11.20 (2.88)	
Retired	136 (7.7%)	11.90 (2.93)	
Students	219 (12.4%)	11.55 (2.76)	
**Income**			
< 10,000 THB	198 (11.2%)	12.44 (2.80)^a^	< 0.001**
10,001–30,000 THB	749 (42.3%)	11.62 (2.95)^b^	
30,001–50,000 THB	514 (29.0%)	11.30 (2.58)^a,c^	
> 50,001 THB	309 (17.5%)	12.83 (2.96)^b,c^	
**Sufficiency of income**			
Sufficient and surplus to save	979 (55.3%)	11.91 (2.82)	0.002*
Sufficient without surplus	636 (35.9%)	11.54 (2.85)^a^	
Not enough, not in debt	82 (4.6%)	12.71 (3.05)^a^	
Not enough, in debt	73 (4.1%)	12.36 (3.52)	
**Health related behaviors**			
**Smoking tobacco**			
Non smoker	1626 (91.9%)	11.85 (2.90)	0.356
Current smoker	61 (3.4%)	11.46 (2.64)	
Former smoker	83 (4.7%)	11.75 (2.80)	
**Drinking alcohol**			
No alcohol	1093 (61.8%)	11.83 (2.90)	0.162
< 3 days/week	618 (34.9%)	11.88 (2.88)	
> 3 days/week	59 (3.3%)	11.31 (2.73)	

*Note:* ^a,b,c^All pairwise comparisons were significant denoted with identical superscript letters in the group. ^d^Mann–Whitney *U* test or Kruskal–Wallis test, testing for distribution across the groups.

Abbreviation: SD, standard deviation.

* *p* value; statistically significant at *p* < 0.05

** *p* value; statistically significant at *p* < 0.001.

### 3.2 Health‐Related Behaviors of the Cohort

Fewer than 10% were either current or former smokers. Most participants reported no alcohol consumption, with fewer than 5% of participants reporting frequent alcohol use (Table 1).

### 3.3 Oral Health‐Related Practices of the Cohort

Over two‐thirds reported regular dental visits. Nearly 90% brushed twice daily, but fewer than one‐third brushed for 2 min or more. About one‐quarter followed the Modified Bass technique, while nearly half combined scrubbing and vertical motions. Most used manual toothbrushes (89.2%), with bristle texture commonly soft or medium. Interdental cleaning was reported by about 60%, most often using dental floss. Among floss users, 77.7% cleaned every interproximal area and 52% curved the floss around the tooth (Table 2).

**Table 2:** Oral health‐related practices of the participants (*n* = 1770).

Oral health‐related practices	*n* (%)	Total ALPHA‐K scores	
		Mean (SD)	*p*‐value^e^
**Dental visit**			
Regularly	1163 (65.7%)	11.91 (2.97)	0.158
Having dental problems	587 (33.2%)	11.71 (2.71)	
Never	20 (1.1%)	11.20 (2.53)	
**Frequency of tooth brushing**			
< 2 times/day	42 (2.4%)	13.26 (3.20)^a^	< 0.001**
2 times/day	1590 (89.8%)	11.69 (2.82)^a,b^	
> 2 times/day	138 (7.8%)	13.00 (3.10)^b^	
**Duration of tooth brushing**			
< 2 min	1250 (70.6%)	11.72 (2.77)	< 0.001**
≥ 2 min	520 (29.4%)	12.10 (3.13)	
**Brushing techniques**			
Scrub	38 (2.1%)	11.71 (2.47)	< 0.001**
Up and down	86 (4.9%)	11.97 (3.03)^a^	
Scrub and up and down	865 (48.9%)	11.75 (2.83)^c^	
Modified bass	456 (25.8%)	12.44 (3.17)^b,c^	
Non pattern	325 (18.4%)	11.18 (2.43)^a,b^	
**Toothbrush types**			
Manual toothbrush	1579 (89.2%)	11.93 (2.87)	0.024*
Electric toothbrush	191 (10.8%)	11.07 (2.92)	
**Texture of toothbrush**			
Extra soft	284 (16%)	12.69 (3.27)^a,c^	< 0.001**
Soft	736 (41.6%)	11.99 (2.91)^b,c^	
Medium	740 (41.8%)	11.36 (2.61)^a,b^	
Hard	10 (0.6%)	11.20 (2.15)	
**Interdental cleaning**			
Use	1060 (59.9%)	12.12 (2.99)	0.009*
Do not use	710 (40.1%)	11.42 (2.67)	
**Interdental device**			
Do not use	710 (40.1%)	11.43 (2.67)^a^	< 0.001**
Floss	779 (44.0%)	12.14 (2.97)^a,d^	
Interdental brushes	27 (1.5%)	13.15 (3.09)^b^	
Toothpick	79 (4.5%)	11.00 (2.51)^b,c,d^	
Oral irrigation	15 (0.8%)	12.73 (2.84)	
Floss and others	160 (9.0%)	12.20 (3.21)^c^	
**Frequency of flossing**			
Do not floss	831 (46.9%)	11.47 (2.69)^a^	0.008*
Everyday	489 (27.6%)	12.09 (3.05)	
Every 2–3 days	308 (17.4%)	12.01 (3.02)	
Once a week	81 (4.6%)	12.44 (2.98)	
Less than once a week	61 (3.4%)	13.00 (2.52)^a^	
**Flossing area**			
Do not floss	831 (46.9%)	11.47 (2.69)^a,b^	0.029*
Every proximal area	730 (41.2%)	12.14 (3.07)^a^	
Only area where food particles stuck	209 (11.8%)	12.21 (2.79)^b^	
**Type of dental floss**			
Do not floss	831 (46.9%)	11.47 (2.69)^a^	< 0.001**
Handheld floss	600 (33.9%)	12.69 (3.22)^a,b^	
Floss holder	339 (19.2%)	11.29 (2.37)^b^	
**Floss curving around each tooth**			
Do not floss	831 (46.9%)	11.47 (2.69)^a^	< 0.001**
Yes	518 (29.3%)	12.66 (3.31)^a,b^	
No	421 (23.8%)	11.53 (2.46)^b^	

*Note:* ^a,b,c,d^All pairwise comparison were significant denoted with identical superscript letters in the group. ^e^Mann–Whitney *U* test or Kruskal–Wallis test, testing for distribution across the groups.

Abbreviation: SD, standard deviation.

* *p* value; significant at *p* < 0.05

** *p* value; significant at *p* < 0.001.

### 3.4 Periodontal Health Knowledge of the Cohort

The mean ALPHA‐K score was 11.83 (2.88), corresponding to 59.2% of the total score, with a median of 12 (IQR 4, range 4–20). Most (85%) correctly identified gum disease can lead to loss while 83% recognized smoking as risk factors. However, major misconceptions persisted: 80% believed flossing is mainly for removing food particles, 65% thought tooth loosening is a natural part of ageing, and 64% believed fluoride prevents gum disease (Table 3).

**Table 3:** Percentage of correct answers for each item of the ALPHA‐K.

Questions	Correct answers	% of correct answers
**1. Dental plaque is the primary cause of gum disease.**	**Yes**	**82%**
2. Minor bleeding on gum line when brushing your teeth is normal.	No	58%
3. If your gums bleed every time when brushing, it is a sign of gum disease.	Yes	80%
4. If there is bleeding when brushing, you should avoid brushing that area.	No	45%
5. If there is bleeding when flossing, you should avoid flossing that area.	No	40%
**6. Gum disease can lead to tooth loss.**	**Yes**	**85%**
7. Teeth loosen naturally as you get older.	No	39%
8. Tooth loss happens naturally as you get older.	No	35%
9. Using a hard‐bristled toothbrush cleans your teeth better than a soft‐bristled one.	No	68%
10. Flossing can reduce the risk of gum disease.	Yes	81%
11. The main goal of flossing is to remove food particles that are stuck between your teeth.	No	20%
12. Flossing causes gaps between your teeth.	No	77%
13. Using a toothpick is adequate for cleaning in between teeth.	No	82%
14. Fluoride can prevent gum disease.	No	36%
15. Herbs in toothpaste can treat gum disease.	No	49%
16. Gargling salt water can treat gum disease.	No	46%
17. Mouthwash can remove tartar.	No	48%
18. Professional dental cleaning can thin your teeth surface.	No	70%
19. Diabetic patients are at a higher risk of gum disease than others.	Yes	62%
**20. Smokers are at higher risk of gum disease than others.**	**Yes**	**83%**

*Note:* Bold = top 3 correct answers, highlight = top 3 misunderstandings.

### 3.5 Association Between the Demographic Data and the ALPHA‐K Scores

Significant differences in knowledge scores were observed across several demographic variables. Middle‐aged adults (40–59 years) achieved higher ALPHA‐K scores than younger and older groups. Women scored significantly higher than men, and participants with postgraduate education outperformed those with a bachelor's degree or lower. Urban residents demonstrated higher knowledge of the disease and etiology than rural residents. Interestingly, both the lowest‐income (< 10,000 THB) and highest‐income (> 50,000 THB) groups achieved higher scores than middle‐income groups, with those reporting insufficient income but no debt also performing better than those with adequate income but no surplus. Significant differences in the foregoing parameters were not found for employment status, smoking, or alcohol consumption (Table 1).

### 3.6 Association Between Oral Health‐Related Practices and the ALPHA‐K Scores

Significant positive associations were found between oral health knowledge scores and several optimal practices: brushing for at least 2 min, employing the Modified Bass technique, using a manual toothbrush with extra‐soft bristles, and regular interdental cleaning. Among interdental aid users, those using interdental brushes scored highest, whereas toothpick users scored significantly lower. Finally, mastery of specific flossing techniques, completing all proximal areas, using a handheld flosser, and proper wrapping technique, were each significantly associated with increased ALPHA‐K scores (Table 2).

### 3.7 Determinants of Periodontal Health Knowledge Based Upon the Total ALPHA‐K Scores

EFA confirmed sampling adequacy (KMO = 0.833; Bartlett's test *p* < 0.001) and identified five components (Table 4): (1) flossing practices; (2) brushing practices and economic background; (3) income sufficiency and gender; (4) education and age; and (5) brushing technique and frequency. In multiple regression analysis (Table 5), higher ALPHA‐K scores were significantly associated with flossing practices (*β* = 0.273, *p* < 0.001), brushing/economic context (*β* = 0.500, *p* < 0.001), and education/age (*β* = 0.352, *p* < 0.001). Brushing/economic context showed the strongest association, underscoring the influence of brushing habits and socioeconomic conditions on periodontal health knowledge.

**Table 4:** Rotated component matrix.

Variable	Factor loading				
	1	2	3	4	5
Flossing area	0.916				
Type of dental floss	0.911				
Floss curving around each tooth	0.910				
Interdental device	−0.910				
Frequency of flossing	0.846				
Interdental device	0.554				
Duration of tooth brushing		0.716			
Place of living		−0.687			
Texture of toothbrush		−0.532			
Toothbrush types		0.342			0.273
Income		0.269	−0.0774		
Sufficiency of income			0.768		
Gender			0.396		
Education				0.748	
Age				0.738	
Brushing techniques		−0.295			0.717
Frequency of tooth brushing					0.590

*Note:* Rotation matrix: Varimax with Kaiser nomination.

**Table 5:** Multiple linear regression analysis. Dependent variable: Total ALPHA‐K score.

Independent variables	Unstandardized coefficients			Standardized coefficients *β*		*t*		*p* value
	*β*	S. E.						
Factor loading 1	0.273	0.67		0.095		4.086		< 0.001*
Factor loading 2	0.500	0.67		0.173		7.492		< 0.001*
Factor loading 3	0.100	0.67		0.350		1.492		0.136
Factor loading 4	0.352	0.67		0.122		5.274		< 0.001*
Factor loading 5	−0.073	0.67		−0.025		−1.087		0.277

* *p* value; statistically significant at *p* < 0.001.

## 4 Discussion

This study, conducted for the first time in a large Thai adult population, offers critical insights into the state of their periodontal health knowledge, which were characterized by a moderate overall level of awareness and the persistence of significant misconceptions. The mean ALPHA‐K score of approximately 60% indicates substantial room for improvement. This was comparable to the 63% reported among non‐dental undergraduate students prior to education (Thongchotchat et al. 2025), but lower than the 71% observed among returning patients at the dental school's periodontal clinic, where greater exposure to professional advice is provided (Bodhidatta et al. 2025). While most participants recognized dental plaque as the primary cause of gingivitis and established risk factors like smoking and diabetes, significant misunderstandings were evident. For instance, many believed that flossing is primarily for removing food debris, and tooth loss are inevitable accompaniments of ageing, and fluoride directly prevents gum disease. These specific knowledge gaps likely hinder the adoption of effective self‐care practices and underscore a clear need for more targeted public education.

Our findings on the socioeconomic determinants of knowledge are consistent with the international literature. Similar to studies from Germany, China and Romania, lower income and rural residence were associated with poorer periodontal knowledge (Deinzer et al. 2009; Zhao et al. 2019; Jin et al. 2025; Wang 2024; Chisnoiu et al. 2022). Education also emerged as a strong predictor with higher educational achievements linked to better ALPHA‐K scores reinforcing the well‐established connection between educational attainment and oral health literacy outcomes (Deinzer et al. 2009; El‐Qaderi and Quteish Ta'ani 2004; Mårtensson et al. 2006; Lin et al. 2001). Another interesting finding from this study was the relatively higher knowledge of the higher‐ and lowest‐income group compared with some middle‐income cohorts. Higher knowledge among higher‐income individuals likely benefits from better access to dental care and health information, while those with low‐income may have greater exposure to public health campaigns that successfully reached the former population group, underscoring the potential impact of well‐directed interventions.

Knowledge of periodontal disease was also strongly associated with oral hygiene practices (Kaira et al. 2012; Shirolkar et al. 2022). Higher ALPHA‐K scores were linked to recommended practices such as brushing for at least 2 min, using the Modified Bass technique, and selecting extra‐soft bristled toothbrushes. This reinforces that effective oral hygiene depends not only brushing frequency but also on correct technique, duration, and tool selection. The interesting observation that manual toothbrush users scored higher than electric toothbrush users is consistent with another study (Wang and Jiang 2020) and may reflect accessibility and socioeconomic issues within the Thai context where manual brushes are more common and affordable. It is tempting to hypothesize that individuals using manual brushes consciously compensate with more meticulous technique, whereas electric brush users may over‐rely on the device itself, potentially neglecting the proper brushing technique.

Flossing practices were particularly discriminant and notable. Participants who performed meticulous flossing (i.e., flossing every proximal area, used handheld floss, and wrapped the floss around the tooth) scored significantly higher. In contrast, those who relying on toothpicks, an ineffective substitute for interproximal plaque removal, had lower knowledge scores. These findings align with reports from China (Zhao et al. 2019; Kwan and Williams 1999), Nepal (Aryal et al. 2021), and Nigeria (Ehizele et al. 2012), where similar misconceptions about the purpose of flossing (plaque removal vs. food dislodgement) and the inevitability of tooth loss were prevalent. Rectifying these myths through targeted education is essential for improving preventive practices.

Factor analysis further highlighted distinct domains that underpin periodontal knowledge. Flossing practices, brushing/economic context, and education/age emerged as independent predictors of knowledge, with brushing/economic context showing the strongest effect. Consistent with this, a previous study reported that individuals with favorable oral hygiene practices, such as frequent brushing, longer brushing duration, and regular flossing had significantly higher ALPHA‐K scores (Thongchotchat et al. 2025). This suggests that better practices are linked with greater knowledge, underscoring the importance of targeting those with poor habits to close the knowledge–practice gap.

Taken together, our findings emphasize the urgent need for tailored, multi‐faceted educational interventions in Thailand to modify oral health related practices. Public health strategies should focus on dispelling misconceptions, promoting evidence‐based practices such as brushing for at least 2 min and daily interdental cleaning, and improving access to affordable hygiene aids (European Federation of Periodontology 2015; American Dental Association 2024). Integrating oral health promotion into school curricula, community programs, and primary care services could effectively reach vulnerable populations, particularly those in rural areas with lower educational attainment. International experience confirm that such comprehensive approaches can reduce knowledge gaps and improve periodontal outcomes at a population‐level (Jürgensen et al. 2012; Watt and Petersen 2012; Watt 2005; Petersen 2009).

Despite being the largest Thai study of this nature to date our investigation has a few limitations. The online survey format may underrepresent older adults and those with limited digital literacy potentially affecting generalizability. Because the survey was distributed through an open, nationwide online approach, the total number of individuals who received the survey link could not be determined; therefore, a response rate could not be calculated. Self‐reported practices are susceptible to recall and social desirability bias, while the cross‐sectional design precludes causal inference. Moreover, the absence of clinical periodontal assessments means one cannot directly link knowledge and reported practices with health outcomes. Future research should include clinical examinations and employ probability‐based sampling to improve generalizability.

## 5 Conclusion

Clear disparities were evident across demographic and socioeconomic groups, with vulnerable populations such as rural residents, those with lower income, and individuals with inadequate brushing duration or preference for hard bristles, as being at particular risk. To address these gaps public health programs should prioritize targeted educational interventions to correct specific misconceptions, especially regarding the purpose of flossing and the inevitability of tooth loss, while reinforcing effective oral hygiene practices. A multi‐sectoral approach that strengthens knowledge through schools, community outreach, and integration into primary care is essential for improving periodontal health outcomes. Ultimately addressing these gaps will not only promote better oral hygiene practices but also contribute to reducing health inequalities and improving overall well‑being in the Thai population.

## Author Contributions

Mana Naratippakorn contributed to methodology, formal analysis, visualization, and writing – original draft of the article. Paswach Wiriyakijja contributed to the conceptualization, methodology, and critical revision of the manuscript. Dissara Akkarasrisawad contributed to the data curation, investigation, and final approval of manuscript. Nirinya Raoprajong contributed to the data curation, investigation, and final approval of manuscript. Pirada Itthipolchai contributed to the data curation, investigation, and final approval of manuscript. Vivat Thongchotchat contributed to data analysis and critical revision of manuscript. Pimchanok Sutthiboonyapan contributed to the conceptualization, methodology, funding acquisition, and critical revision of the manuscript. All authors contributed to the final approval of the version to be submitted.

## Ethics Statement

The present study adhered to the Declaration of Helsinki and received approval from the Ethics Committee at the Faculty of Dentistry, Chulalongkorn University (approval number: 2023‐123, date of approval: December 1, 2023).

## Consent

All participants gave consent before starting the questionnaires.

## Conflicts of Interest

The authors declare no conflicts of interest.

## Data Availability

Data are available upon request from the authors.
